# A High-Performance Synthetic Jet Piezoelectric Air Pump with Petal-Shaped Channel

**DOI:** 10.3390/s22093227

**Published:** 2022-04-22

**Authors:** Xingqi Li, Xiaopeng Liu, Luntao Dong, Xiaodong Sun, Huajie Tang, Guojun Liu

**Affiliations:** 1 College of Mechanical and Aerospace Engineering, Jilin University, Changchun 130025, China; xingqi20@mails.jlu.edu.cn (X.L.); liuxp20@mails.jlu.edu.cn (X.L.); james.dong@es-precision.com (L.D.); hjtang18@mails.jlu.edu.cn (H.T.); 2 College of Communication Engineering, Jilin University, Changchun 130025, China; sunxd@jlu.edu.cn

**Keywords:** piezoelectric air pump, synthetic jet, petal-shaped channel, high performance, orthogonal tests

## Abstract

The synthetic jet piezoelectric air pump is a potential miniature device for electronic cooling. In order to improve the performance of the device, a small-sized synthetic jet piezoelectric air pump is proposed in this work, which is mainly characterized by petal-shaped inlet channels. First, the structure and working principle of the piezoelectric vibrator and the proposed pump are analyzed. Then, three synthetic jet piezoelectric air pumps with different inlet channels are compared. These inlets are the direct channels, the diffuser/nozzle channels, and the petal-shaped channels, respectively. Furthermore, the performance of the synthetic jet piezoelectric air pump with the petal-shaped inlet channels is optimized by orthogonal tests. Finally, the simulation was used to investigate the heat dissipation capability of the synthetic jet piezoelectric pump. The experimental results show that among the three inlet channels, the petal-shaped channel can greatly improve the performance of the pump. The unoptimized pump with petal-shaped channels has a maximum flow rate of 1.8929 L/min at 100 V, 3.9 kHz. Additionally, the optimized pump with petal-shaped channels reaches a maximum flow rate of 3.0088 L/min at 100 V, 3.7 kHz, which is 58.95% higher than the unoptimized one. The proposed synthetic jet piezoelectric air pump greatly improves the output performance and has the advantages of simple structure, low cost, and easy integration. The convective heat transfer coefficient of the synthetic jet piezoelectric pump is 28.8 $W/\left(m^{2}\cdot {}^{\circ}C\right)$, which can prove that the device has a better heat dissipation capability.

## 1. Introduction

The microfluidics system, demonstrating high precision, small size, and low power consumption, has received more attention in recent years during the rapid development of micro-electromechanical systems (MEMS). Thus, many micro-devices such as microvalves [1], micromixers [2], and micropumps [3] have been developed. Among them, the micropump is one of the vital components for microfluidics in the form of a new type of actuator. In addition, with advances in piezoelectric drive technology, piezoelectric micropumps are used to manipulate small fluid volumes to achieve a wide range of applications in the field of biological detection [4], chemical analysis [5], cooling [6], and fuel supply [7].

The synthetic jet piezoelectric air pump is a kind of piezoelectric micropump, and its working principle is based on the synthetic jet mechanism. It has promising applications in the field of heat dissipation and cooling [8,9]. Over the recent two decades, a variety of improved structures have been developed for synthetic jet piezoelectric air pumps [10,11]. These studies mainly focus on multi-vibrator and multi-chamber structures. Jalilvand et al. proposed a dual-vibrator synthetic jet actuator, which produces an airflow velocity of more than 7 m/s, an improvement of 3.5 times than the single-vibrator one [12]. Liu et al. designed a dual-chamber synthetic jet piezoelectric air pump with a flow rate of 2.43 L/min, an increase of 15.7% compared to the single-chamber pump [13,14]. Researchers continuously optimized the structure of the jet holes in several ways, such as in combination with Tesla or diffuser/nozzle elements, to reduce the reverse flow of the fluid [15]. Tran et al. provided a synthetic jet piezoelectric air pump with a Tesla coupling nozzle, which can reach a flow rate of 14 mL/min [16]. Further, Fathima et al. studied a valveless micropump combined with a diffuser/nozzle that achieved a maximum flow rate of 31.15 mL/min [17].

Based on the above analysis, scholars have conducted various studies with remarked results, but there are still many parts to be studied. For example, few studies have been reported on the small size of synthetic jet piezoelectric air pumps and their inlet channel structures. To this end, a sub-size synthetic jet piezoelectric air pump with petal-shaped channels is reasonably proposed in this work. Pumps with three different inlets were compared experimentally to demonstrate the superiority of the proposed pump with petal-shaped channels. Subsequently, systematic orthogonal tests were used to optimize the synthetic jet piezoelectric air pump with petal channels to achieve optimal output performance. Finally, the researchers explored the heat dissipation capability of the synthetic jet piezoelectric pump by the means of simulation.

## 2. Design and Principle

### 2.1. Design and Principle of the Piezoelectric Vibrator

A novel synthetic jet piezoelectric air pump with petal-shaped inlet channels is proposed in this work. Two circular piezoelectric vibrators are used as the driving part of the pump, one of which has a circular hole in the center. The piezoelectric vibrator is formed by bonding a ceramic (PZT-5) and a copper substrate. Table 1 lists typical parameters of the piezoelectric vibrator.

When a sinusoidal signal is applied to the piezoelectric vibrator, the piezoelectric ceramic produces an inverse piezoelectric effect, which causes the piezoelectric vibrator to periodically deform up and down. Thus, the change of the chamber volume forms a pressure difference effect, which realizes the unidirectional continuous flow of the fluid.

The deformation of piezoelectric vibrators can be considered a thin plate problem with small deflection. According to the plate and shell theory [18], the displacement of the thin circle plate with small deflection can be expressed as:

(1) $$w\left(r\right)=-\frac{P}{8\pi D}\left[\frac{1}{2}\left(a^{2}-r^{2}\right)+r^{2}\ln\frac{r}{a}\right]$$

Thus, the displacement of the piezoelectric vibrator can be expressed as:

(2) $$w\left(r\right)=A\left[1-\frac{r^{2}}{a^{2}}+\frac{2r^{2}}{a^{2}}\ln\left(\frac{r}{a}\right)\right]$$

Here, *r* is the distance from any point on the piezoelectric vibrator to the center, *a* is the radius of the piezoelectric vibrator, and *A* is the maximum displacement of the piezoelectric vibrator.

When the piezoelectric vibrator is driven by a sinusoidal voltage, its displacement can be expressed as:

(3) $$w\left(r,t\right)=\frac{K}{2}\sin\left(2\pi ft\right)\left[1-\frac{r^{2}}{a^{2}}+\frac{2r^{2}}{a^{2}}\ln\left(\frac{r}{a}\right)\right]$$

where, *K* is the peak-to-peak displacement at the center. Equation (3) can be used to simulate the vibrator amplitude of the synthetic jet piezoelectric pump.

### 2.2. Design and Principle of the Synthetic Jet Piezoelectric Air Pump

**Figure 1:**
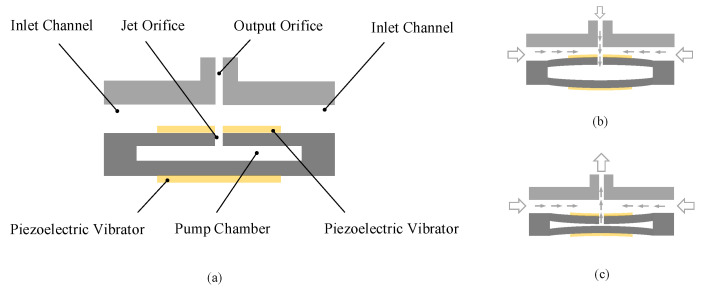
Working process of the pump: (**a**) static state; (**b**) supply process; (**c**) pump process.

The operation of periods for the piezoelectric pump can be divided into two parts, which are the supply and pump process. The researchers applied a sinusoidal excitation voltage with the same frequency and no phase difference to two piezoelectric vibrators in a periodic process. Figure 1a displays a static schematic of the proposed pump, which is not working at this time. Figure 1b shows the supply process, the two piezoelectric vibrators are bent away from each other. At this time, the gas will be sucked into the pump chamber through the orifice due to the structural characteristics. Instead, Figure 1c reveals a pump process in which the two piezoelectric vibrators bend towards each other. The reduced volume of the pump chamber increases the pressure inside the chamber. Then, the gas is subjected to the role of shear action at the orifice in the chamber, and the gas in the cavity block is pumped from the jet hole. Further, the synthetic jet piezoelectric pump repeats the working process of the above cycle. It is worth noting that when the synthetic jet pump is in the supply mode again, due to the principle of synthetic jet, the jet gas ejected during the pumping process is already far away from the hole, so it will not be affected by the supply process.

The formation of the synthetic jet is based on the appropriate structural parameters and excitation conditions; thus, it is vital to explore the phenomenon of synthetic. In 1998, Smith and Glezer studied this special phenomenon [19,20,21]. In order to facilitate the study, a large number of parameters are defined as well as the establishment of the “Slug” model according to the literature.

One of the important dimensionless parameters for synthetic jets is dimensionless stroke length, which is defined by $L=\frac{L_{0}}{d_{0}}$. $L_{0}$ is stroke length of the whole cycle of a synthetic jet, $d_{0}$ is jet hole diameter.

Note that $\frac{\bar{U_{0}}}{fd_{0}}$ is closely related to the inverse of the Strouhal number. $\bar{U_{0}}$ is the average velocity scale for the synthetic jet, ω=2πf is the radian frequency of the vibrator.

(4) $$L=\frac{L_{0}}{d_{0}}=\frac{\bar{U_{0}}T}{d_{0}}=\frac{\bar{U_{0}}}{fd_{0}}\sim \frac{1}{S_{t}}=\frac{\bar{U_{0}}}{\omega d_{0}}=\frac{\bar{U_{0}}d_{0}/v}{\omega d_{0}{}^{2}/v}=\frac{Re}{S^{2}}$$

where, according to Reynolds number (*Re*) and Stokes number (*S*) in Equation (4) and, it is obvious *L* has a similar form to the Strouhal number.

According to Equation (3), because the volume of the pump cavity of the synthetic jet piezoelectric pump is conserved, the velocity of the corresponding point can be obtained by its corresponding displacement:

(5) $$u\left(r,t\right)=\frac{dw\left(r,t\right)}{dt}=\pi fK\;\cos\left(2\pi ft\right)\;\left[1-\frac{r^{2}}{a^{2}}+\frac{2r^{2}}{a^{2}}\ln\left(\frac{r}{a}\right)\right]$$

Thus, the instantaneous mass flow rate in the orifice area can be expressed as:

(6) $$\dot{Q_{0}}\left(t\right)=\rho u_{0}\left(t\right)A=\rho \int_{0}^{r_{c}}u\left(r,t\right)\cdot 2\pi rdr=\frac{\pi^{2}}{16}\rho fKd_{c}{}^{2}\sin\left(2\pi ft\right)$$

where $d_{c}$ is the diameter of piezoelectric vibrator in Equation (6), and its value is twice the value of *a*.

Thus, the instantaneous velocity at the jet hole can be expressed as:

(7) $$u_{0}\left(t\right)=\frac{\dot{Q_{0}}\left(t\right)}{\rho A}=\frac{\pi }{4}fK\left(\frac{d_{c}{}^{2}}{d_{0}{}^{2}}\right)\sin\left(2\pi ft\right)$$

Meanwhile, the average velocity of the synthetic jet in a cycle can be defined as:

(8) $$\bar{U_{0}}=\frac{1}{T}\overset{T/2}{\underset{0}{\int }}u_{0}\left(t\right)dt=\frac{1}{4}Kf\left(\frac{d_{c}{}^{2}}{d_{0}{}^{2}}\right)$$

Then,

(9) $$L=\frac{L_{0}}{d_{0}}=\frac{\bar{U_{0}}T}{d_{0}}=\frac{1}{4d_{0}}K\left(\frac{d_{c}{}^{2}}{d_{0}{}^{2}}\right)$$

In order for the synthetic jet to be formed, Equation (4) needs to be satisfied:

(10) $$\frac{Re}{S^{2}}>C$$

Here, *S* is the Stokes number. *C* is the jet formation constant, depending on orifice shape, the radius of curvature, etc. For axisymmetric synthetic jet phenomena, *C* = 0.16 [22]. The specific parameters of the actuator are shown in Table 2, L=5.65 mm, $L_{0}=5.09\;\mathrm{mm},$ $S_{t}=0.90$, $\frac{Re}{S^{2}}=1.11>0.16$. In this case, the proposed piezoelectric pump can form a synthetic jet.

**Figure 2:**
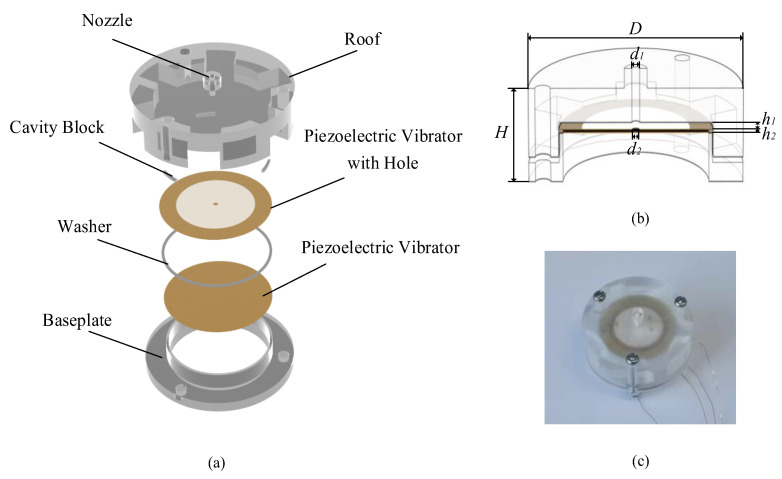
Model and prototype of the piezoelectric micropump: (**a**) explosive view; (**b**) cross-sectional view; (**c**) prototype.

Figure 2a reveals a schematic diagram of the proposed synthetic jet structure and micropump. Figure 2b illustrates the cross-sectional view of the synthetic jet piezoelectric air pump. With a diameter of 28 mm and a thickness of 10 mm, these piezoelectric micropumps are relatively small in size for existing designs. The piezoelectric pump is mainly composed of the roof, the cavity block, washer, circular piezoelectric vibrators, and baseplate.

**Figure 3:**
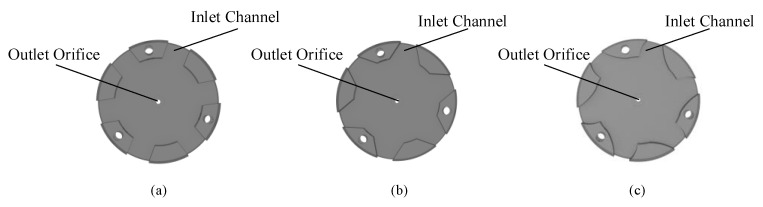
The roof with different inlet channel: (**a**) direct channel; (**b**) diffuser/nozzle channel; (**c**) petal-shaped channel.

The cavity block makes a gap between the piezoelectric vibrator with a hole and the roof. The pump chamber is formed by the combination of the washer and two different piezoelectric vibrators. The height of the pump chamber is determined by the thickness of the gasket. There are three positioning holes that were arranged on the roof and baseplate, and the whole structure is held together by three bolts. At once, the piezoelectric air pump adopted a double shaker structure as the driven element. By adjusting the excitation frequency applied on the two shakers, the volume change rate of the pump chamber can be maximized to achieve the maximum flow rate of the fluid. The specific parameters of the piezoelectric pump structure are shown in Table 2. There are three different inlet channels in the roof which were designed as Figure 3, the gas can enter the piezoelectric air pump through the channel, such as the direct channel, the diffuser/nozzle channel, and the petal-shaped channel. The researchers believe that the piezoelectric air pump with a diffuser/nozzle channel can deliver gas more efficiently than the piezoelectric air pump with a direct channel. The petal-shaped channel is similar in structure to the diffuser/nozzle channel, the difference is that there is a curvature of the channel, which can effectively assist the supply and pump of gas.

## 3. Prototype Fabrication and Experimental Platform

**Figure 4:**
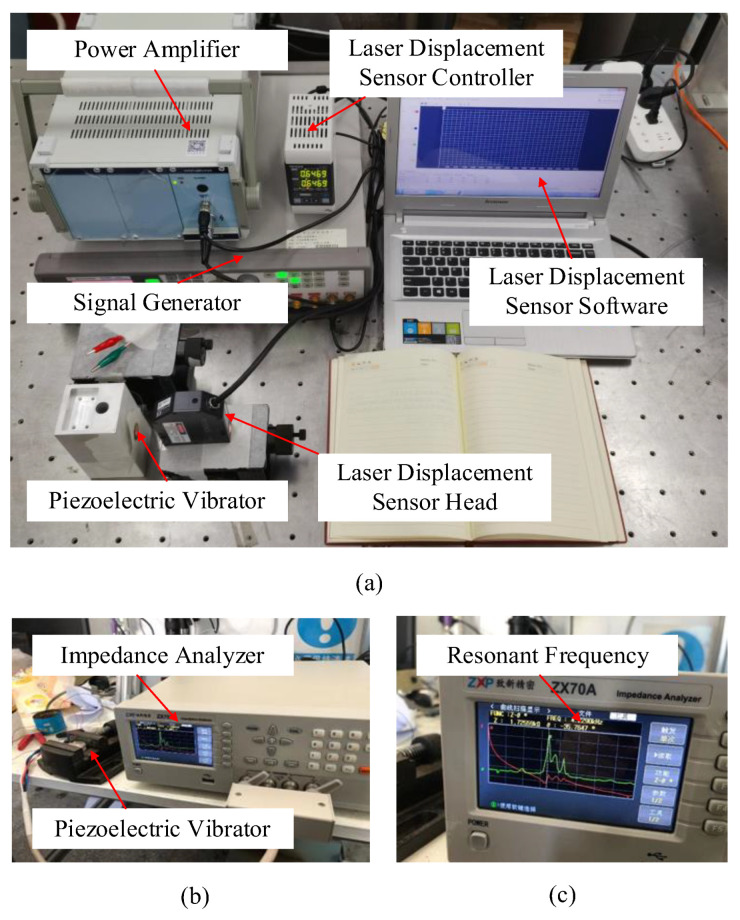
Test platforms of piezoelectric vibrators: (**a**) test platform of amplitude; (**b**) sweep test platform; (**c**) impedance analysis waveform diagram.

In order to obtain the accurate resonant frequency, we measured the amplitude of piezoelectric vibrators at different frequencies with a laser micrometer (Keyence, Osaka, Japan, LK-G30) and swept the vibrators with an impedance analyzer (ZXP, Changzhou, China, ZX704). The experimental platforms are shown in Figure 4a,b, respectively.

**Figure 5:**
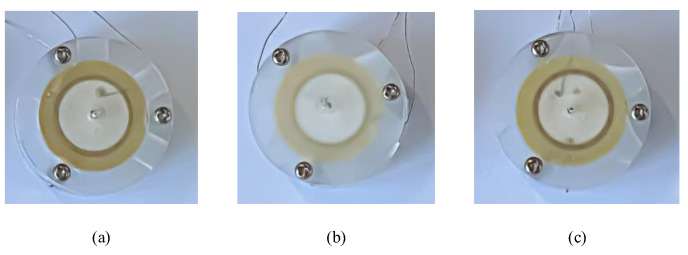
The prototype of the synthetic jet piezoelectric air pump with different inlet channel: (**a**). with direct channel; (**b**) with diffuser/nozzle channel; (**c**) with petal-shaped channel.

**Figure 6:**
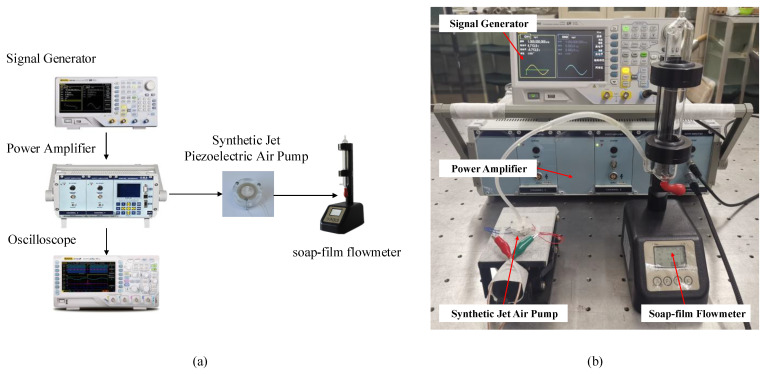
Test of synthetic jet piezoelectric air pump: (**a**). the test system of the synthetic jet piezoelectric air pump; (**b**). test platforms of synthetic jet piezoelectric air pump.

In this paper, the effects of different inlet channels on the performance of synthetic jet piezoelectric pumps are verified by comparative experiments. To increase the reliability of the comparison experiments, the dimensions of the inlet channels of the three piezoelectric pumps were limited. The three different inlet channels have the same channel width at the middle position. Meanwhile, the entrance position and the exit position of the diffuser/nozzle channel and the petal-shaped channel have the same width. The three different kinds of prototypes were fabricated and tested. During the process of fabricating the prototypes, the upper roof of the prototypes needs to have a certain thickness to maintain the stability of the overall structure. Meanwhile, the three cavity blocks, which form the height of the cavity, should be placed on the same side under the condition that the three bolts are used to fix the synthetic jet piezoelectric pump. Figure 5 and Figure 6 are the prototype photos and test platform, respectively. The sinusoidal signals are given by the signal generator (Rigol, Beijing, China, DG4062), which magnify by a power amplifier (Coremorrow, Harbin, China, E01.A3). An oscilloscope (Rigol, Beijing, China, DS1102Z-E) is used to measure the accuracy of the output sinusoidal signals. The output flow rate of the synthetic jet piezoelectric air pump can be measured by a soap-film flowmeter (Labsanli, Shenzhen, China, ZM-101B). These are all the components of the overall test structure.

## 4. Results and Discussion

### 4.1. Simulation and Experiment of the Piezoelectric Vibrator

**Figure 7:**
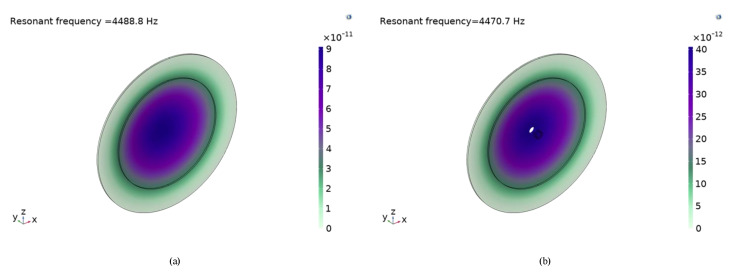
The first−order modes of the vibrators: (**a**) of the vibrator; (**b**) of the vibrator with hole.

In order to further explore the characteristics of piezoelectric vibrators, the simulation software COMSOL multiphysics and experimental verification were used for analysis. The piezoelectric vibrator has different modes at different natural frequencies, so it is vital to carry out the modal simulation, which can obtain the appropriate mode of the vibration. The natural frequency under the appropriate mode can be used as the working frequency. Table 3 shows the resonant frequencies of the first four orders of the circular piezoelectric vibrators. From the mode shapes shown in simulation Figure 7, the first-order shape of the vibrator is arched form, the maximum amplitude is in the most central position of the piezoelectric vibrator under this situation. In the case of the first mode, the pump chamber volume change rate of the piezoelectric pump is the largest, which is the best order shape for the piezoelectric pump, and its corresponding natural frequency is 4.4888 kHz. The piezoelectric vibrator with holes has a similar situation, and its corresponding natural frequency is 4.4707 kHz. The above two natural frequencies can be used as reference frequencies in experiments.

**Figure 8:**
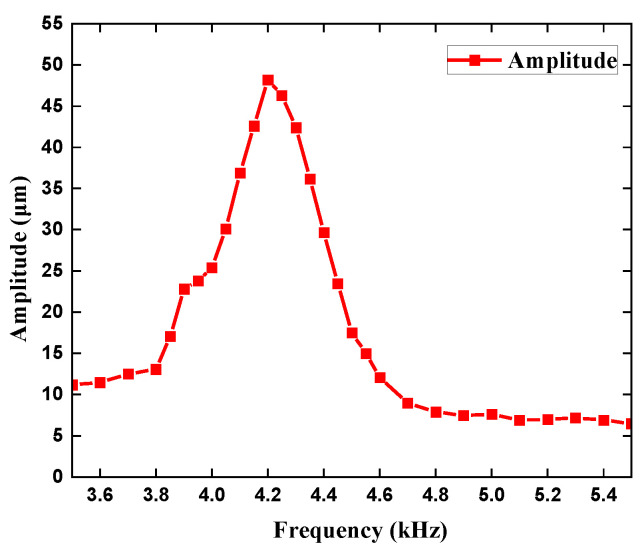
The amplitude curve of the piezoelectric vibrator.

As shown by the frequency-amplitude Figure 8, it is obvious to find that the amplitude of the two different vibrators has the same trend with frequency, which increases and then decreases. The maximum amplitude of the piezoelectric vibrator is 48.2 μm at 4.2 kHz. The maximum amplitude of the piezoelectric vibrator with a hole is also 48.2 μm at 4.2 kHz. In general, the experimental results are similar to the simulation values. The determination of the natural frequency of the piezoelectric vibrator plays an important part in the selection of simulation parameters in the follow-up simulation of the synthetic jet piezoelectric air pump. In addition, the specific result is shown in Figure 8. Scanning in the frequency range of 0.05 kHz−10 kHz, the resonance frequency of the piezoelectric pump is about 4.23 kHz, which is roughly the same as the simulation result and measurement result. The specific value of the resonance frequency can be found in Figure 4c.

### 4.2. Experiment Test of the Synthetic Jet Piezoelectric Air Pump

**Figure 9:**
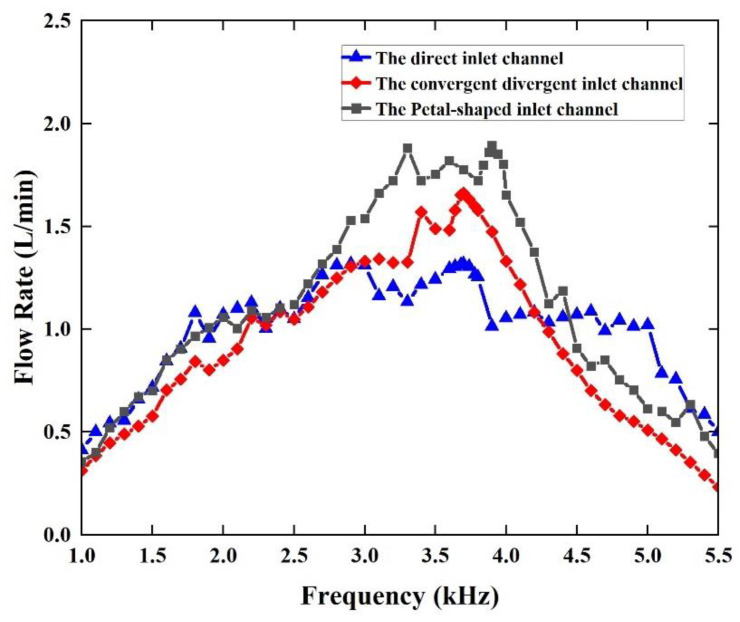
Flowrate curve of different prototypes.

The flow curves of three different synthetic jet piezoelectric pumps at a frequency of 1 kHz−5 kHz were drawn in Figure 9. The influence of synthetic jet piezoelectric pumps’ inlet channel structure on their performance can be investigated by the trend in Figure 9. The flow rate of the synthetic jet pump with the direct channel is 1.3172 L/min at 100 V and 3.7 kHz. The flow rate of the synthetic jet pump with the diffuser/nozzle channel is 1.6618 L/min at 100 V and 3.7 kHz. The synthetic jet pump with the petal-shaped channel has the best performance, which has a maximum flow rate of 1.8929 L/min at 100 V and 3.9 kHz.

### 4.3. Structure Optimization

The structure of the synthetic jet piezoelectric air pump with the petal-shaped channel is optimized to obtain the optimal structure. The important factors affecting the performance of the piezoelectric pump were identified at first. As shown in Figure 2b, there are four main parameters that affect the gas output performance of the piezoelectric air pump, which are the diameter of the air outlet ($d_{1}$), the diameter of the jet hole ($d_{2}$), the height of the cavity ($h_{1}$), and the height of the chamber ($h_{2}$). Considering that the test involves four factors, the workload of the test becomes large if the control variable method is adopted for the test. In order to achieve results equivalent to a large number of full-scale tests with a minimum number of trials, the orthogonal test design is undoubtedly a better choice. It is necessary to point out that orthogonal experimental designs are used to explore the influence of main parameters on piezoelectric synthetic jet velocity. Table 4 is the orthogonal factor level table.

Based on the problem of four factors and five levels, an orthogonal table of format L_25_(56) is chosen. Only 25 sets of experiments are needed to represent all possible combinations under this situation. Furthermore, because the experiment is a four factors and five levels problem, the given data can not fill the corresponding orthogonal table. This situation leads to the existence of an error list. The existence of the list does not affect the experimental results. Then, the researchers conducted experiments in accordance with the designed orthogonal table. In this paper, the performance index is the gas output flow rate. The importance of the influence of various factors on the gas output flow is discussed comprehensively by range analysis. The orthogonal table is shown in Table 5, which illustrates different cases and results. $K_{i}$ is the sum value of the test result at level i, which is divided by the number of level i, and R is extremum difference. The order of the magnitude of the factors affecting the synthetic jet piezoelectric air pump can be obtained by the analysis method called range analysis, which uses the magnitude of the *R* value to determine the importance of each factor. In addition, the optimal combination of the synthetic jet piezoelectric air pump can be confirmed by the value of $K_{i}$. The experimental results show that the factors affecting the performance of the pump are, in order of importance, the diameter of the outlet (*d*_1_), the height of the cavity (*h*_1_), the diameter of the jet hole (*d*_2_), and the height of the chamber (*h*_2_). Meanwhile, the optimal combination of the pump is $d_{1}=1.2\;\mathrm{mm}$, $d_{2}=1.0\;\mathrm{mm}$, $h_{1}=0.8\;\mathrm{mm}$, $h_{2}=0.3\;\mathrm{mm}$ by comparing the value of R.

**Figure 10:**
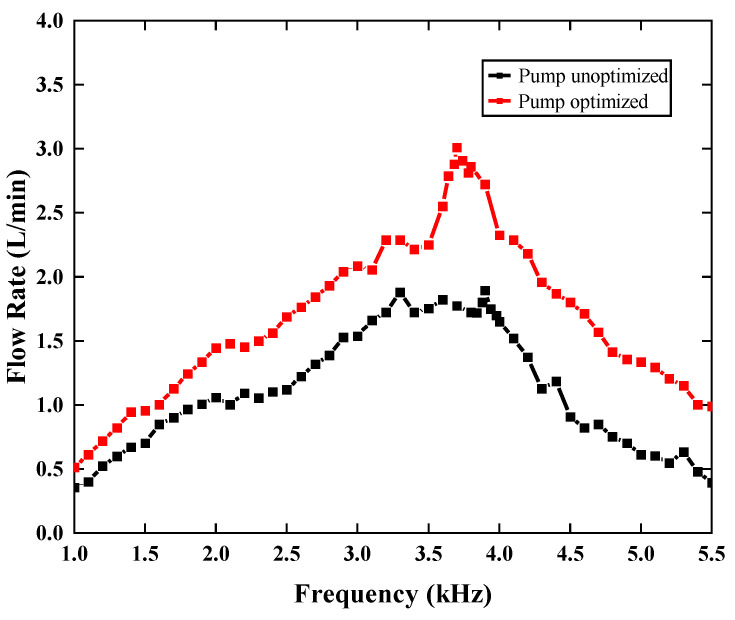
Flowrate curve of unoptimized pump and optimized pump.

The performance of both unoptimized piezoelectric pumps and optimized piezoelectric pumps are shown in Figure 10. It is not difficult to find the performance of the optimized piezoelectric pump is better than the unoptimized one. The maximum flow of the piezoelectric pump with the optimal structure is 3.0088 L/min at 100 V and 3.7 kHz, which is 58.95% higher than before optimization. According to the corresponding frequency, the optimal frequency of the synthetic jet piezoelectric pump is between 3.5 kHz and 4.2 kHz. In fact, because the system of the piezoelectric pump affects the resonant frequency of the piezoelectric vibrator, the corresponding frequency of the pump is not exactly the same as the result given in Section 4.1.

### 4.4. Simulation and Discussion of the Heat Transfer Characteristics of the Pump

Analyzing the preceding context, it is obvious to conclude that the new synthetic jet piezoelectric pump studied in the paper has a better operating performance. The results of the optimization experiments above can be used to discuss the heat transfer performance of the synthetic jet piezoelectric pump. In this section, the researchers combine and study the synthetic jet and radiator, meanwhile, the combination of which has some research value in the field of engineering heat transfer in electronic devices. In order to save the computational resources, the simulation software COMSOL Multiphysics can be used to simulate the radiator as well as the heat source.

**Figure 11:**
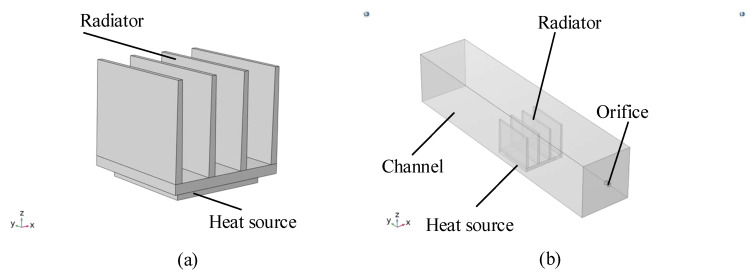
Simulation model: (**a**). natural convection heat dissipation; (**b**). synthetic jet heat dissipation.

**Figure 12:**
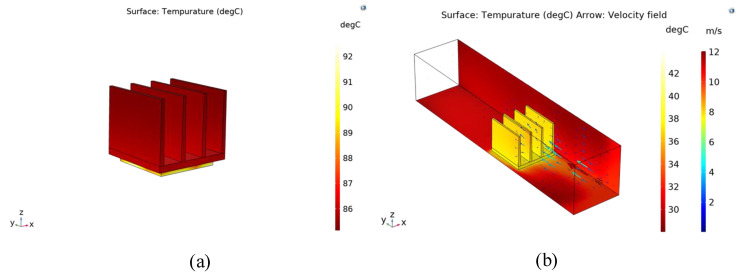
Temperature nephogram: (**a**). natural convection heat dissipation; (**b**). synthetic jet heat dissipation.

The specific simulation process is divided into two parts. The first part is a simulation using convection cooling boundary conditions on the radiator boundary. This means that the heat distribution of the radiator is calculated based on the natural convective heat transfer coefficient. The empirical value of the natural convection heat transfer coefficient is 10 $W/\left(m^{2}\cdot {}^{\circ}C\right)$. Figure 11a shows the model of the heat sink combined with the heat source. In the second part, the channels are introduced into the simulation model. The heat distribution of the radiator is obtained by calculating the heat balance between the heat source, the heat sink, and the channel. In Figure 11b, it is shown that the radiator is installed in a rectangular channel, and the heat source is placed below the heat sink, which emits heat of 2 W. The fluid enters through the nozzle and leaves through the exit of the channel. The specific simulation results are shown in Figure 12.

The maximum temperature of the radiator is about 93 °C, as can be seen from the temperature nephogram of natural convection heat dissipation. The maximum temperature of the heat sink is about 43 °C as shown in the temperature nephogram of the synthetic jet heat sink. In contrast, the synthetic jet heat dissipation showed a better heat dissipation effect.

(11) $$h=\frac{Q}{A\left(T_{avg}-T_{e}\right)}$$

Meanwhile, the convective heat transfer coefficient can be used as a measure of thermal performance, which is defined by Equation (11). Q is the heat power, A is the area of the radiating surface, and $T_{avg}$ is the average surface temperature of the heat resource, and $T_{e}$ is the environmental temperature. The convective heat transfer coefficient of this piezoelectric pump can be estimated from the simulation results as about 28.8 $W/\left(m^{2}\cdot {}^{\circ}C\right)$. The above results can prove that this piezoelectric pump has a good cooling capacity.

## 5. Conclusions

In this paper, a novel synthetic jet piezoelectric air pump with the petal-shaped channels was presented. The piezoelectric micropump has a diameter of 28 mm and a thickness of 10 mm. The comparative tests were designed to study the performance of the proposed synthetic jet piezoelectric pump, and the optimization experiments were designed to optimize the structure of the pump. The output flow of the piezoelectric pump was analyzed as an index. The results of the experiment are summarized as follows:

(1) It is reasonable to understand that the optimal working frequency of the synthetic jet piezoelectric pump is between 3.5 kHz and 4.2 kHz, which is near the first-order frequency of the piezoelectric vibrator (Figure 9 and Figure 10) (Table 5).
(2) The performance advantage of the synthetic jet piezoelectric pump with a petal-shaped inlet channel is proven by comparative experiments. Compared with the synthetic jet piezoelectric pump with the direct channel, the flow performance is improved by 43.7%. Compared with the synthetic jet piezoelectric pump with the diffuser/nozzle channel, the flow performance is improved by 13.9% (Figure 9).
(3) Meanwhile, the primary and secondary order of parameters affecting the performance of the synthetic jet piezoelectric pump is obtained by means of variance analysis of the orthogonal test, which is the diameter of the air outlet ($d_{1}$), the height of the cavity ($h_{1}$), the diameter of the jet hole ($d_{2}$), and the height of the chamber ($h_{2}$) (Table 5).
(4) According to the optimization experiment based on orthogonal design, the parameters of the synthetic jet piezoelectric pump with the best structure are $d_{1}=1.2\;\mathrm{mm}$, $d_{2}=1.0\;\mathrm{mm}$, $h_{1}=0.8\;\mathrm{mm}$, $h_{2}=0.3\;\mathrm{mm}$. At the 100 V, 3.7 kHz, the maximum flow rate of the synthetic jet piezoelectric pump with petal-shaped inlet channels is achieved 3.0088 L/min, which is 58.95% higher than optimized before (Figure 10).
(5) After understanding the operating performance of a synthetic jet piezoelectric pump, the cooling performance of this piezoelectric pump is discussed. The researchers obtained the convective heat transfer coefficient of this device to be 28.8 $W/\left(m^{2}\cdot {}^{\circ}C\right)$ by means of simulation, demonstrating the cooling capabilities of this device (Figure 12).

The proposed novel synthetic jet piezoelectric air pump has achieved a breakthrough in small size and large flow rate. At the same time, there are several benefits demonstrated by piezoelectric pumps, including simple structure, cost-effectiveness, and wide application range. In general, the innovation of its structure has a strong practical significance for the development of small device heat dissipation mode and the research of small device heat dissipation.

## Figures and Tables

**Table 1:** Related parameters of the piezoelectric vibrator.

Type	Material	Diameter (mm)	Thickness (mm)	Density (kg·m^−3^)	Modulus of Elasticity (GPa)	Poisson’s Ratio
Ceramic	PZT-5	14	0.2	7600	63	0.32
Substrate	Copper	20	0.2	8920	118	0.35

**Table 2:** Structural parameters of the piezoelectric air pump.

Parameter	Symbol	Value (mm)
Diameter of piezoelectric pump	*D*	28
Height of piezoelectric pump	*H*	10
Diameter of air outlet	$d_{1}$	1.0
Diameter of jet hole	$d_{2}$	0.9
Height of cavity	$h_{1}$	0.5
Height of pump chamber	$h_{2}$	0.2

**Table 3:** The first four order resonant frequencies of the piezoelectric vibrators.

Order	Resonant Frequency of Circular Piezoelectric Vibrator (Hz)	Resonant Frequency of Circular Piezoelectric Vibrator with Hole (Hz)
1	4488.8	4470.7
2	9555.6	9553.9
3	9657.1	9655.7
4	15,332	15,306

**Table 4:** Orthogonal factor level table.

Level/Factors	$d_{1}(\mathrm{mm})$	$d_{2}(\mathrm{mm})$	$h_{1}(\mathrm{mm})$	$h_{2}(\mathrm{mm})$
1	0.8	0.6	0.4	0.1
2	0.9	0.7	0.5	0.2
3	1.0	0.8	0.6	0.3
4	1.1	0.9	0.7	0.4
5	1.2	1.0	0.8	0.5

**Table 5:** Orthogonal table and results.

Group Number	Factors						Result Q (L/min)	Corresponding Frequency (kHz)
	$d_{1}(\mathrm{mm})$	$d_{2}(\mathrm{mm})$	$h_{1}(\mathrm{mm})$	$h_{2}(\mathrm{mm})$	Error List 1	Error List 2		
1	0.8	0.6	0.4	0.1	1	1	1.0547	3.9
2	0.8	0.7	0.5	0.2	2	2	1.2992	4.1
3	0.8	0.8	0.6	0.3	3	3	1.7536	3.9
4	0.8	0.9	0.7	0.4	4	4	1.1253	3.5
5	0.8	1	0.8	0.5	5	5	1.8682	3.7
6	0.9	0.6	0.5	0.3	4	5	1.3294	3.5
7	0.9	0.7	0.6	0.4	5	1	1.4583	3.9
8	0.9	0.8	0.7	0.5	1	2	1.9313	3.6
9	0.9	0.9	0.8	0.1	2	3	2.1987	3.9
10	0.9	1	0.4	0.2	3	4	1.4436	3.5
11	1	0.6	0.6	0.5	2	4	1.624	3.6
12	1	0.7	0.7	0.1	3	5	1.8805	3.7
13	1	0.8	0.8	0.2	4	1	2.0417	4.1
14	1	0.9	0.4	0.3	5	2	2.0564	3.6
15	1	1	0.5	0.4	1	3	2.2507	3.2
16	1.1	0.6	0.7	0.2	5	3	1.6618	4.2
17	1.1	0.7	0.8	0.3	1	4	3.0088	3.8
18	1.1	0.8	0.4	0.4	2	5	1.6715	3.9
19	1.1	0.9	0.5	0.5	3	1	2.1491	3.8
20	1.1	1	0.6	0.1	4	3	2.7751	3.7
21	1.2	0.6	0.8	0.4	3	2	2.9775	3.8
22	1.2	0.7	0.4	0.5	4	3	2.1819	3.6
23	1.2	0.8	0.5	0.1	5	4	2.0864	4.0
24	1.2	0.9	0.6	0.2	1	5	2.5985	4.1
25	1.2	1	0.7	0.3	2	1	2.4855	3.9
K1	1.420	1.729	1.682	1.999	2.169	1.838		
K2	1.672	1.966	1.823	1.809	1.856	2.066		
K3	1.971	1.897	2.042	2.127	2.041	2.137		
K4	2.253	2.026	1.817	1.897	1.891	1.858		
K5	2.466	2.165	2.419	1.951	1.826	1.870		
R	1.046	0.436	0.737	0.318	0.343	0.299		

## Data Availability

Not applicable.

## Nomenclature

P	The concentrated force on a plate at the center of a circle plate, [N].
r	The length between any point on the circle plate and the center of a circle plate, [mm].
a	The radius of the circle plate, [mm].
A	The maximum displacement of the piezoelectric vibrator, [mm].
K	The peak-to peak displacement at the center, [mm].
L	The stroke length, [mm].
$L_{0}$	The stroke length of the whole cycle of a synthetic jet, [mm].
$\bar{U_{0}}$	The average velocity scale for the synthetic jet, [L/s].
$d_{0}$	The jet hole diameter of synthetic jet actuator, [mm].
$S_{t}$	Strouhal number
ω	The radian frequency of vibrator, [1/s].
v	Kinematic viscosity of the fluid, [$m^{2}/s$].
C	Jet formation constant
*T*	One cycle period of a synthetic jet, [s]
Re	Reynolds number
S	Stokes number
$\dot{Q_{0}}$	The instantaneous mass flow rate in orifice area, [kg/s].
$u_{0}$	The instantaneous velocity at the jet hole, [L/s].
$d_{c}$	The diameter of piezoelectric vibrator, [mm].
h	Heat transfer coefficient, $W/\left(m^{2}\cdot {}^{\circ}C\right).$
Q	Heat Power, [W].
A	radiating surface, [$m^{2}]$.
$T_{avg}$	The average surface temperature of the heat resource, [°C].
$T_{e}$	Environment temperature, [°C].
